# Large intra-articular true lipoma of the knee

**DOI:** 10.1186/s12891-019-2484-5

**Published:** 2019-03-18

**Authors:** Jaime Dalla Rosa, José Juan Nogales Zafra

**Affiliations:** 1 Complejo Hospitalario Integral Privado, Avda. Carlos Haya 121, CP: 29010 Málaga, Spain; 2 Chief of Orthoapedic Surgery and Traumatology Department, Complejo Hospitalario Integral Privado, Avda. Carlos Haya 121, CP: 29010 Málaga, Spain

**Keywords:** Lipoma, Knee joint, Arthrotomy, Arthroscopy, Magnetic resonance imaging, Case report

## Abstract

**Background:**

Intra-articular lipomas are rare and very few cases have been reported in the knee. To the best of our knowledge, here we report the largest lipoma to have ever been observed in the knee. It is crucial to avoid the misdiagnose of lipoma arborescens, which is associated with degenerative joint disease. Lipoma is a homogeneous, ovoid, adipose tissue tumor that is contained within a fibrous capsule and not associated with previous disease.

**Case presentation:**

A 48-year-old male, with a soft-tissue tumor on the superomedial aspect of the right knee. Magnetic resonance imaging (MRI) revealed an intra-articular lipoma. The mass was resected by means of a limited arthrotomy.

**Conclusions:**

Knee lipoma is an extremely rare disease that must be diagnosed by MRI. Where possible, it should be resolved by arthroscopic resection.

## Background

To the best of our knowledge, this is the largest lipoma presented within the knee joint, at least in the literature [1–19].

Although lipomas are the most common type of soft-tissue tumor, intra-articular examples are extremely rare. This contrasts starkly with lipoma arborescens, which is a well-established clinical entity that develops in joints secondary to a degenerative process rather than as a neoplasm [20]. As mentioned already, intra-articular lipomas are extremely rare, with the most frequent location being the knee, although cases have also been described in the hip, lumbar spine, elbow, shoulder and wrist [21]. He we present the clinical case of a patient with an intra-articular lipoma in his knee.

## Case presentation

A 48-year-old male with no relevant medical history was referred to our service from another hospital with a soft-tissue mass on his right knee. The patient noticed the mass several years earlier, but due to its size and the absence of symptoms, he did not seek medical assistance. The mass progressively increased in size and deep flexion became uncomfortable. No history of previous trauma was reported.

**Fig. 1:**
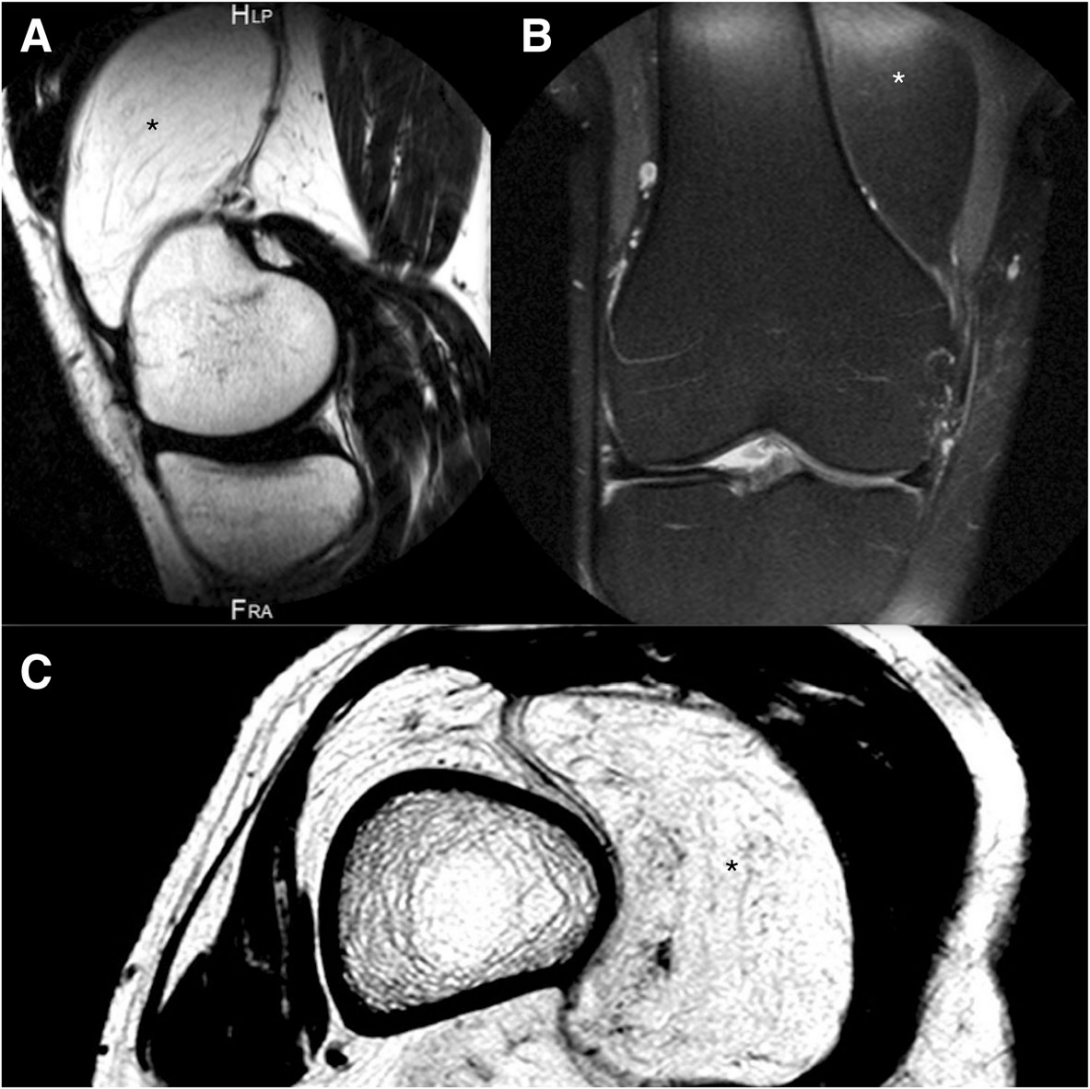
MRI of the knee shows an intraarticular lipoma (*): **a**): T1-w image in Sagital view. **b**: T2-w FS image in Coronal view. **c** T1-w image in Axial view at the level of the suprapatellar pouch

Physical examination revealed a mass of soft tissue in the superomedial aspect of the right knee. Upon palpation, the mass was soft, nontender and adhered to deep planes. There was no localized temperature increase or joint effusion. The range of movement was 0–110° with no mechanical symptoms, although deep flexion was painful. There was no evidence of muscular atrophy. Complementary tests and diagnostic imaging: Blood test results were within normal limits. Weight-bearing X-rays of the knees were normal, presenting only radiolucent soft tissue and no signs of degenerative joint disease. T1-weighted (T1-w) and T2-weighted (T2-w) MRI sequences revealed a soft-tissue mass with high signal intensity containing linear structures of low signal intensity (Fig. 1a, b and c) that were isointense with the subcutaneous fat. The tumor was located in the medial suprapatellar bursa but crossed into the lateral region and occupied the patellofemoral joint in extension.

**Fig. 2:**
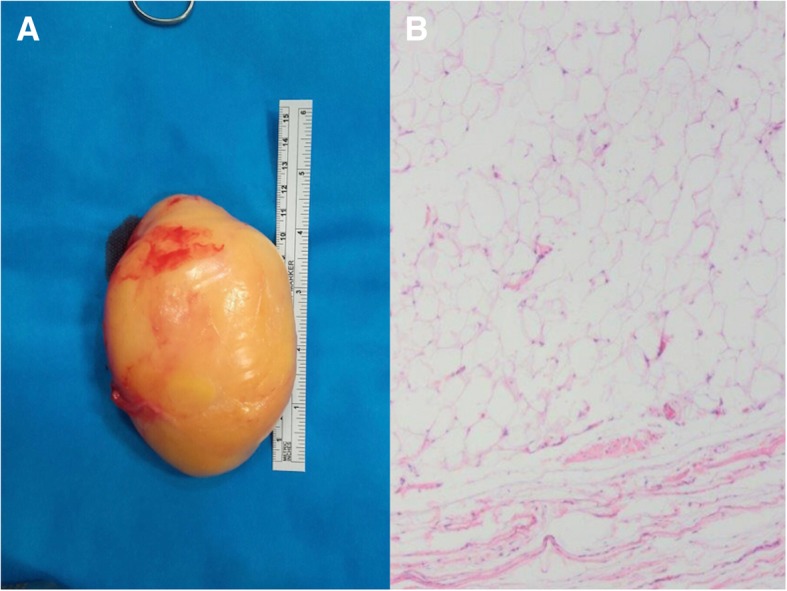
**a** Tumor after resection with ruler for scale. **b** Microscope image showing mature adipocytes without an atypical nucleus separated by fibrous septa

Treatment: A limited medial parapatellar arthrotomy excision was performed (by using only the superior part of the midline longitudinal approach). We encountered a soft ovoid mass measuring about 13 × 8.5 cm, with a fibrous capsule (Fig. 2a), that was not adhered to deep planes, although it was anchored by a fibrous tract to the suprapatellar bursa; we cauterized this tract. The lateral portion of the lesion passed through the patellofemoral joint into a lateral location. We performed a complete resection of the lesion and sent it to the pathology department. They confirmed it was a true intra-articular lipoma, observing typical images of mature adipocytes without an atypical nucleus and separated by fibrous septa (Fig. 2b).

Outcome and follow-up: The case coursed with a favorable postoperative evolution. The surgical site incision healed without complications, although a keloid scar formed on the skin. The patient suffered from postoperative rigidity that was resolved with physiotherapy sessions. He was discharged without symptoms and with a complete range of motion. Table 1 shows a timeline of the postoperative evolution.

**Table 1:** Patient visits timeline

Dates	Relevant Past Medical History and Interventions		
	No allergies. No diseases. No previous surgeries.		
Date	Summaries from Initial and Follow-up Visits	Diagnostic Testing (including dates)	Interventions
10/03/2017	1st Outpatient visit	MRI	Weight bearing Xrays
10/14/2017	2° visit		Inclusion to surgery
10/17/2017	Operation Room		Surgery
11/01/2017	Staples removal		Prescription of physiotherapy
11/28/2017	3rd Visit	Clinical Exam: Full range of motion. No pain. No effusion.	Revision
03/28/2018	4th visit	Asymptomatic	Return to play
04/17/2018	5th visit	Case report	Informed Consent

Lipomas are commonplace soft-tissue tumors, and can be found anywhere in the body [22]. Intra-articular lipomas, however, are a very rare entity, with approximately 27 cases published to date, of which 19 affected the knee [23]. Initially, intra-articular lipoma can be difficult to diagnose, especially when it is small and there is no apparent lesion discernible on conventional radiographs. If a lesion can be identified, it appears as an area of well-defined radiolucency. The next step in the study of this lesion is MRI, which is considered the tool of choice for the detection of intra-articular masses and meniscal-ligamentous lesions [24]. It manifests as a high intense signal in T1-w and T2-w sequences, which is analogous to the signal intensity of the subcutaneous fatty tissue. However, lipoma can also appear with nonspecific characteristics on MRI, such as a signal intensity analogous to fluid, that is thought to be due to mucoid degeneration [25].

Differential diagnosis should be made to rule out lipoma arborescens, intra-articular liposarcoma, pigmented villonodular synovitis (PVNS) and Hoffa’s disease [26].

Macroscopically, lipoma arborescens has the appearance of villous synovial proliferation of fatty tissue. It is also associated with some clinical conditions such as previous trauma, osteoarthritis and other chronic inflammatory conditions (e.g., rheumatoid arthritis and psoriatic arthritis), whereas, intra-articular lipoma occurs de novo without any previous history. On MRI, lipoma arborescens produces “hairy” projections in the synovium with a high signal intensity in T1-w and T2-w sequences, that is saturated on Short-Tau Inversion Recovery Images (STIR) [27].

Low-grade liposarcoma affects middle-aged people. It usually presents as a painless, slow-growing, locally aggressive tumor that rarely metastasizes. Intra-articular liposarcoma is rare. On MRI, it appears as a large lesion with thick septa, accompanied by non-lipomatous soft tissue with a low fatty component.

PVNS is a rare intra-articular lesion that affects the synovial membrane of joints and tendon sheaths. MRI tends to reveal a low signal intensity on T1-w and T2-w sequences with “blooming effect” [28], which is due to the magnetic susceptibility of hemosiderin deposits.

Hoffa’s disease refers to impingement of infrapatellar fat, first described by Hoffa in 1904. Infrapatellar fat becomes hypertrophic due to previous trauma. In this case, MRI will show a mass of low signal intensity in T1-w and T2-w images, attributable to subacute or chronic fibrosis. On T2-w images, there may also be an increase in signal intensity in cases of inflammation or hemorrhage. Hoffa’s disease is occasionally accompanied by ossification. However, unlike intra-articular lipoma, it is rarely found in the suprapatellar bursa or intercondylar region [29].

Histopathologically, intra-articular lipoma is consisted on mature adipocytes covered with a synovial membrane and may also contain a vascular fibrous septum. That is why it is a true neoplasm of uncertain etiology. The natural history of the disease has not been studied in depth, although it is known that it grows slowly and follows a silent clinical course until the symptons appearance due to a space-occupying lesion.

The gold-standard treatment has not yet been established for intra-articular lipoma. Arthroscopic excision has been performed as well as open arthrotomy. There have been no recurrences of the lesion following arthroscopic excision in previous studies, which suggests this treatment is valid so long as it is practicable. Arthroscopy did not seem to be an option in our case given the large size of the patient’s lesion, so we deemed limited arthrotomy to be a much more realistic option.

## Conclusions

Clinical examination is essential in the diagnosis of the lesion. MRI is primordial for both differential diagnosis and preoperative planning. Excision can be performed by open arthrotomy or arthroscopy, depending on the size of the lesion.

## Abbreviations

MRI: Magnetic resonance imaging
PVNS: Pigmented villonodular synovitis
STIR: Short-Tau Inversion Recovery
T1-w: T1-weighted
T2-w FS: T2-weighted with fat saturation
T2-w: T2-weighted
